# Medicinal leech therapy in neurosurgical practice

**Published:** 2012-11

**Authors:** Hamed Hanif, Mohsen Nouri, Abbas Amirjamshidi

**Affiliations:** ^a^Department of Neurosurgery, Sina Hospital, Tehran Iran.

## Abstract

Hirudo Medicinalis application in medicine has long been a matter of investigation since Hippocrates. The analgesic and anti-coagulant substances in the leeches’ saliva are used in treatment of venous thrombosis and varicose veins, arthralgia, rheumatologic diseases, low back and cervical pains. Aeromonas infections, excessive bleeding, anemia, and allergic reactions are among the most common complications reported with leech therapy.

The present study, reports leech therapy in two patients: one for sciatalgia and the other for deep venous thrombosis. Furthermore, a comprehensive review of the literature on different aspects of leech therapy with special focus on its neurosurgical applications and related dysfunctions is presented.

**Keywords:**

Leech therapy, Neurosurgical, Medicinal


					Volume 4, Suppl. 1 Nov 2012
Publisher: Kermanshah University of Medical Sciences
URL: http://www.jivresearch.org
Copyright:
Congress of Iranian Neurosurgeons, 14-16 November 2012, Kermanshah, Iran
Paper No. 72
Medicinal leech therapy in neurosurgical practice
Hamed Hanif a
, Mohsen Nouri a
, Abbas Amirjamshidi a,*
a
Department of Neurosurgery, Sina Hospital, Tehran Iran.
Abstract:
Abstract:
Hirudo Medicinalis application in medicine has long been a matter of investigation since Hippocrates. The analgesic
and anti-coagulant substances in the leeches' saliva are used in treatment of venous thrombosis and varicose veins,
arthralgia, rheumatologic diseases, low back and cervical pains. Aeromonas infections, excessive bleeding, anemia,
and allergic reactions are among the most common complications reported with leech therapy.
The present study, reports leech therapy in two patients: one for sciatalgia and the other for deep venous thrombosis.
Furthermore, a comprehensive review of the literature on different aspects of leech therapy with special focus on its
neurosurgical applications and related dysfunctions is presented.
Key words:
Leech therapy, Neurosurgical, Medicinal
* Corresponding Author at:
Abbas Amirjamshidi: Department of Neurosurgery, Sina Hospital, Tehran Iran. Tel: 09121329864,Email: abamirjamshidi@yahoo.com, (Amirjamshidi
A.).
Volume 4, Suppl. 1 Nov 2012
Publisher: Kermanshah University of Medical Sciences
URL: http://www.jivresearch.org
Copyright:
Congress of Iranian Neurosurgeons, 14-16 November 2012, Kermanshah, Iran

				

